# Pinin protects astrocytes from cell death after acute ischemic stroke via maintenance of mitochondrial anti-apoptotic and bioenergetics functions

**DOI:** 10.1186/s12929-019-0538-5

**Published:** 2019-06-05

**Authors:** Sujira Mukda, Ching-Yi Tsai, Steve Leu, Jenq-Lin Yang, Samuel H. H. Chan

**Affiliations:** 1 Research Center for Neuroscience, Institute of Molecular Biosciences, Mahidol University, 25/25 Phuttamonthon 4 Road, Salaya, Nakhon Pathom, 73170 Thailand; 2 Institute for Translational Research in Biomedicine, Kaohsiung Chang Gung Memorial Hospital, 123 Dapi Rd, Kaohsiung, 83301 Taiwan

**Keywords:** Middle cerebral artery occlusion, Oxygen-glucose deprivation, Primary cultured astrocytes, Cytotoxic edema, Peri-infarct area

## Abstract

**Background:**

Stroke is the second most common cause of deaths worldwide. After an ischemic stroke, the proliferated reactive astrocytes in the peri-infarct areas play a beneficial role in neuronal survival. As such, astrocytes have gradually become a target for neuroprotection in stroke. The present study assessed the hypothesis that Pinin (Pnn), originally identified as a nuclear and desmosome-associated protein and is now known to possess anti-apoptotic capacity, protects astrocytes from cell death after ischemic stroke and delineated the underlying mechanisms.

**Methods:**

In in vivo experiments, adult male Sprague-Dawley rats (12-week old) were used to induce acute focal cerebral ischemia employing the middle cerebral artery occlusion (MCAO) method. In in vitro experiments, postnatal day 1 (P1) Sprague-Dawley rat pups were used to prepare cultures of primary astrocytes. Oxygen-glucose deprivation (OGD) and re-oxygenation (OGD/R) procedures were employed to mimic the hypoxic-ischemic condition of stroke in those astrocytes.

**Results:**

We found in the peri-infarct area of the ipsilateral cortex and striatum in Sprague-Dawley rats after transient MCAO an increase in Pnn expression that correlated positively with the time-course of infarction as detected by T2-weighted imaging and triphenyltetrazolium chloride staining, augmented number of reactive astrocytes that double-labelled with Pnn as determined by immunofluorescence, and enhanced cytotoxic edema as revealed by diffusion weighted imaging; but mirrored the decreased cleaved caspase-3 as measured by western blot. In an OGD and OGD/R model using primary cultured astrocytes, treatment with *Pnn* siRNA doubled the chance of surviving astrocytes to manifest cell death as revealed by flow cytometry, and blunted activated ERK signaling, reduced Bcl-2 expression and augmented cleaved caspase 3 detected by western blot in the normoxia, OGD or OGD/R group. Gene-knockdown of *Pnn* also impeded the reversal from decline in cell viability, elevation in lactate dehydrogenase leakage and decrease in ATP production in the OGD/R group.

**Conclusion:**

We conclude that the endogenous Pnn participates in neuroprotection after acute ischemic stroke by preserving the viability of astrocytes that survived the ischemic challenge via maintenance of mitochondrial anti-apoptotic and bioenergetics functions.

**Electronic supplementary material:**

The online version of this article (10.1186/s12929-019-0538-5) contains supplementary material, which is available to authorized users.

## Background

Stroke is the second most common cause of mortality worldwide (WHO) [1], with almost 6 million deaths from stroke every year. According to the American Stroke Association [2], a majority (87%) of strokes is ischemic caused by an obstruction within a blood vessel supplying blood to the brain. Although age-standardized rates of stroke mortality have decreased worldwide during the last two decades, the absolute annual numbers of patients who died from stroke or survived but subsisted with the consequences of stroke are increasing [3]. With the burden of stroke becoming a growing global issue, better understanding of this clinical phenomenon is warranted. Whereas ischemic stroke is accompanied by necrosis in the core, apoptosis often occurs in the peri-infarct region (penumbra). Since this apoptotic death is the culprit for lingering pathological consequences [4], preventing its occurrence and understanding its underlying mechanism become a crucial issue.

Pinin (Pnn) was identified in 1992 as a nuclear and desmosome-associated protein [5, 6], and has since been shown to participate in cell-cell adhesion, cancer progression, and regulation of pre-mRNA alternative splicing and export in the nucleus [6–11]. Previous studies indicated that Pnn plays an essential role during mouse development [12–14]; loss of Pnn results in mouse embryonic lethality and cellular apoptosis [12]. Recent studies also demonstrated that Pnn participates in proliferation and metastasis of colorectal, hepatic and ovarian cancer [10, 11, 15]. Work on the role of Pnn in brain functions, however, is wanting.

As the most abundant cell type in the central nervous system, astrocytes participate in maintaining normal brain functions as a structural and functional component of the blood brain barrier and tripartite synapses [16, 17], along with communication with neurons, oligodendrocytes and endothelial cells [18]. After an ischemic stroke, the proliferated reactive astrocytes in the peri-infarct areas play a beneficial role in neuronal survival by restricting the lesion and minimizing the area of inflammation during the acute phase [19, 20]. As such, astrocytes have gradually become a target for neuroprotection in stroke [21–24].

Leu et al. [13] demonstrated that loss of Pnn in cells triggers cellular apoptosis. Yang et al. [11] also reported that over-expression of Pnn attenuates glucose deprivation-induced apoptosis in tumor cells. Given the anti-apoptotic capacity of Pnn and the neuroprotective roles of astrocytes in ischemic stroke, it is conceivable that Pnn may contribute to the latter process by reducing astrocytic cell death induced under ischemic conditions. The present study was undertaken to assess this hypothesis and delineated the underlying mechanisms. Based on corroborative observations from animal and cell models of ischemic stroke, we validated this hypothesis and demonstrated that Pnn preserves the viability of astrocytes that survived the ischemic challenge via maintenance of mitochondrial anti-apoptotic and bioenergetics functions.

## Methods

### Animals

Adult male Sprague-Dawley rats (12-week old; 366–390 g) purchased from BioLASCO, Taiwan, and postnatal day 1 (P1) Sprague-Dawley rat pups from our breeding colony were used. Animals were housed in an AAALAC International-accredited Center for Laboratory Animals, with maintained room temperature (24 ± 1 °C) and 12 h light/12 h dark cycle. Standard laboratory rat chow and tap water were available *ad libitum*. All experimental procedures were approved by the Institutional Animal Care and Use Committee of the Kaohsiung Chang Gung Memorial Hospital (IACUC Number: 2015100501), and were conducted in accordance with the Animal Protection Law set forth by the Council of Agriculture, Taiwan and AAALAC-International *Guide for the Care and Use of Laboratory Animals*.

### Induction of focal cerebral ischemia

We used the middle cerebral artery occlusion (MCAO) method to induce acute focal cerebral ischemia in adult male Sprague-Dawley rats, following published procedures [25–28] with modifications. In brief, under 2% isoflurane anesthesia, a punctate incision with a 25G needle was made in the ventral wall of the left common carotid artery (CCA). A nylon monofilament suture (RWD Life Science; Shenzhen, China), 5 cm in length with a silicone-rubber coated tip (0.43 ± 0.02 mm diameter), was advanced into the CCA lumen towards the MCA via the internal carotid artery. The distance travelled was typically 20 mm. After 90 min of MCAO, the suture was withdrawn, the incision covered using cyanoacrylate glue, and patency of perfusion in the CCA verified. The wound was then closed after recording the end of occlusion time (beginning of reperfusion time). Animals were given 3 ml of normal saline subcutaneously to prevent dehydration, and were continuously monitored for full recovery from anesthesia. Sodium penicillin (10,000 IU; YF Chemical, Taiwan) was given intramuscularly to prevent postoperative infection. As a routine, the effects of 90-min transient MCAO, and 6 h or 24 h after reperfusion (MCAO/R) were evaluated in this study. Animals that received the same surgical procedures under isoflurane anesthesia but without MCAO served as the sham controls. The selection of animals to receive MCAO, MCAO/R or as the sham-controls was completely randomized. Detailed procedures for induction of focal cerebral ischemia are described in Additional file 1: Supplementary materials and methods.

### Magnetic resonance imaging (MRI)

We carried out sequential MRI acquisition using a 9.4 T horizontal-bore animal MR scanning system (Biospec 94/20; Bruker, Ettingen, Germany) in rats under 1.5% isoflurane anesthesia to measure brain infarction and edema. T2-weighted coronal imaging (T2WI) was carried out using multislice turbo rapid acquisition with refocusing echoes (Turbo-RARE) sequence; ImageJ version 1.48v was used to quantify areas of hyperintensity in the acquired images (Additional file 2: Figure S1a). Diffusion weighted imaging (DWI) was performed using DtiEpi sequence on the same spatial brain dimension as in T2WI. ParaVision 5.1 software (Bruker) and MIstar (ver. 3.2.63; Apollo Medical Imaging Technology, Melbourne, Australia) were applied to process the DWI and apparent diffusion coefficient (ADC) maps respectively. The parameters for MRI acquisition were provided in Additional file 1: Supplementary materials and methods.

### Triphenyltetrazolium chloride staining

2,3,5-Triphenyltetrazolium chloride (TTC; Sigma-Aldrich, St. Louis, MO, USA) was dissolved in phosphate-buffered saline (PBS) at 0.05% (w/v) concentration and used immediately for staining. The brain was isolated, sliced into serial 2-mm-thick coronal sections, and incubated in 0.05% TTC solution at 37 °C for 45 min [29]. After staining, the slices were washed in PBS and fixed in 4% paraformaldehyde (Sigma-Aldrich) at 4 °C for 24 h. Quantification of infarct volume was based on the ratio between areas stained in red indicating normal tissue and areas stained in white indicating infarct lesion (Additional file 2: Figure S1b).

### Immunofluorescence staining

As reported previously [30], immunofluorescence staining was carried out using a mouse monoclonal anti-glial fibrillary acidic protein (GFAP) (Thermo Fisher Scientific, Rockford, IL, USA) antibody or rabbit polyclonal anti-Pnn antibody (Sigma-Aldrich). The secondary antibodies used included a goat anti-rabbit IgG conjugated with Alexa Fluor 488 and a goat anti-mouse IgG conjugated with Alexa Fluor 568 (Thermo Fisher Scientific). Viewed under a Fluoview FV1000 laser scanning confocal microscope (Olympus; Tokyo, Japan), immunoreactivity for GFAP exhibited red fluorescence, and Pnn exhibited green fluorescence. ImageJ (version 1.48) was used to quantify GFAP immunoreactivity in the cortical and striatal areas.

### Culture of rat primary astrocytes

Culture of rat primary astrocytes was performed as described previously [31] with modifications. Briefly, the cerebral cortex was aseptically dissected from postnatal day 1 (P1) Sprague-Dawley rat pups and placed in Gibco Minimum Essential Media (MEM; Thermo Fisher Scientific) containing 2 mg/ml trypsin (Sigma-Aldrich). After incubation at 37 °C in the CO_2_ incubator for 30 min, the cortical tissues were mechanically triturated with pipette until they dissociated into single cells. Fetal bovine serum (FBS; 10% final concentration) was then added to stop the activity of trypsin. Cells were seeded into polyethylenimine-coated T75 culture flask at a density of 2 × 10^6^ cells and incubated at 37 °C in the CO_2_ incubator for 3–4 h until the cells attached. On replacement with Dulbecco’s Modified Eagle Medium (DMEM; Thermo Fisher Scientific) that contains high glucose supplemented with N-2 supplement, 10% heat-inactivated FBS and 1% penicillin/streptomycin, cells were incubated at 37 °C in a humidified 5% CO_2_ incubator. After 7–8 days, the confluent cultures were shaken for 30 min to minimize microglia contamination. The remaining primary astrocytes were trypsinized and re-seeded for further experiments.

### Oxygen-glucose deprivation and re-oxygenation

Oxygen-glucose deprivation (OGD) and re-oxygenation (OGD/R) were employed to mimic the hypoxic-ischemic condition of stroke in in vitro experiments. Hypoxic exposure was provided by a Heracell 150i CO_2_ incubator (Thermo Fisher Scientific, Waltham, MA, USA). Glucose-free DMEM was prepared by gassing in the hypoxic chamber with 5% CO_2_, 1% O_2_, and 94% N_2_ for at least 12 h. For the OGD experiment, the culture medium was removed and astrocytes were washed once with pre-warmed (37 °C) PBS. OGD was induced by incubating the primary astrocytes in the pre-gassed glucose-free DMEM medium in a hypoxic chamber with 5% CO_2_, 1% O_2_, and 94% N_2_ for 24 h. The normoxic control cells were incubated at 37 °C in a humidified atmosphere of 95% air/5% CO_2_. Following OGD exposure, the incubation medium was replaced with the conditioned medium and the cultured cells were returned to a CO_2_ incubator at 37 °C. Cells were collected after 24 h of OGD exposure or after 24 h of OGD/R for subsequent experiments. The selection of astrocytes to receive normoxia, OGD or OGD/R was completely randomized.

### siRNA transfection

ON-TARGET *plus* SMART pool for rat Pnn siRNA (Cat# L-100331-02-0050) was obtained from Dharmacon (GE Healthcare, Lafayette, CO, USA). The siRNA was resuspended in 1x siRNA buffer (60 mM KCl, 6 mM HEPES-pH 7.5, and 0.2 mM MgCl_2_) to attend a 20 μM stock concentration. Rat primary astrocytes cultured until reaching 40–50% confluent were transfected with Lipofectamine RNAiMAX (Invitrogen, Carlsbad, CA, USA) according to the manufacturer’s protocol. The RNAi duplex-Lipofectamine RNAiMAX complexes were prepared in Gibco Opti-MEM I Reduced Serum Medium (Thermo Fisher Scientific) and incubated for 5 min at room temperature. The Lipofectamine RNAiMAX was used to transfected the primary astrocytes in 100-mm culture dishes at a final concentration of 10 nM siRNA. After 48 h of incubation at 37 °C in a CO_2_ incubator, siRNA-transfected primary astrocytes were used for OGD and OGD/R studies. For non-specific siRNA control, the ON-TARGET *plus* Non-Targeting Pool (Cat# D-001810-10-20) obtained from Dharmacon was used. The effective concentrations and transfection time for siRNA treatment were determined according to the results presented in Additional file 3: Figure S2.

### WST-1 cell viability assay

Cell viability was evaluated using Cell Proliferation Reagent WST-1 (Roche, Basel, Switzerland) according to the manufacturer’s protocol. Primary cultured astrocytes were seeded into 96-well plates at a density of 5 × 10^4^ cells per well. After replacing the incubation medium with the WST-1 Reagent diluted in a fresh growth medium (1:10), cells were incubated at 37 °C in a CO_2_ incubator for 4 h. Absorbance was then measured at 450 nm with the reference wavelength at 650 nm using a Thermo Scientific Multiskan Spectrum microplate spectrophotometer.

### Lactate dehydrogenase leakage assay

Lactate dehydrogenase (LDH) released from damaged primary astrocytes into the culture medium was determined using a Cytotoxicity Detection Kit^PLUS^ (LDH) kit (Roche) according to the manufacturer’s instructions. The LDH reaction mixture (100 μl) was added to 100 μl of culture medium and incubated for 30 min at room temperature. Stop solution was added and the absorbance was measured at a wavelength of 490 nm using a Thermo Scientific Multiskan Spectrum microplate spectrophotometer.

### Determination of ATP levels

Changes in cellular ATP levels were determined using an ATP Detection Assay Kit (Cayman Chemical, Ann Arbor, MI, USA). Primary astrocytes were lysed with RIPA buffer (50 mM Tris, pH 8.0, 150 mM NaCl, 0.5% sodium deoxycholate, 0.1% SDS), followed by homogenization through sonication and centrifugation at 12,000 x g for 15 min at 4 °C. The supernatants were collected and used for ATP determination. ATP reaction mixture containing luciferase-luciferin buffer (100 μl) was added to 10 μl of cell lysate. Luminescence was measured using a Centro LB 960 Microplate Luminometer (Berthold Technologies, Bad Wildbad, Germany). ATP values were determined from a standard curve and normalized to the protein content of each sample.

### Flow cytometry

To determine cell death status, primary astrocytes were harvested and washed, and stained with the eBioscience Annexin V-FITC Apoptosis Detection Kit (Invitrogen, Carlsbad, CA, USA). For each experiment, cells harvested from OGD or OGD/R group pooled from two dishes were used. The stained cells were analyzed by the Gallios Flow Cytometer (Beckman Coulter, Indianapolis, IN, USA). Annexin V- and propidium iodide (PI)-double negative cells were classified as viable, annexin V-positive and PI-negative as early apoptotic, annexin V- and PI-positive cells as late apoptotic, and annexin V-negative and PI-positive cells as necrotic. Data from the experiments were analyzed by the Kaluza software (Beckman Coulter). Detailed procedures were described in Additional file 1: Supplementary materials and methods and Additional file 4: Figure S3.

### Protein extraction and Western blot analysis

Cortical or striatal tissues from animals, as reported previously [32], were homogenized on ice in a T-PER tissue protein extraction buffer (Thermo Fisher Scientific) that contains protease and phosphatase inhibitors, and centrifuged at 10,000 x g at 4 °C for 10 min. In some experiments, proteins from the cytosolic fraction of the samples were extracted by a commercial kit (Active Motif, Carlsbad, CA, USA). Primary astrocytes, as described previously [33], were lysed in RIPA buffer (50 mM Tris-HCl pH 8.0, 150 mM NaCl, 0.5% sodium deoxycholate, 0.1% SDS and 1% protease and phosphatase inhibitor), followed by homogenization through sonication and centrifugation at 12,000 x g for 15 min at 4 °C. In all cases, the supernatant was collected, and the concentration of total proteins was determined by the Pierce BCA Protein Assay Kit (Thermo Fisher Scientific).

Protein samples (50 μg) were electrophoresed on a 7.5% or 12.5% SDS-PAGE gels and then transferred onto a polyvinylidenedifluoride (PVDF) membrane. The membrane was incubated overnight at 4 °C with primary antibodies: anti-Pnn (Abcam, Cambridge, MA, USA), anti-ERK (Cell Signaling Technology, Danvers, MA, USA), anti-phospho-ERK (Cell Signaling Technology), anti-Bcl2 (Santa Cruz Biotechnology, Dallas, TX, USA), anti-cleaved caspase-3 (Cell Signaling Technology), or anti-β-actin (Merck Millipore, Darmstadt, Germany). The membrane was subsequently incubated with a specific horseradish peroxidase (HRP)-conjugated secondary antibody for 90 min, visualized through enhanced chemiluminescence using the UVP BioSpectrum 600 Imaging System (Analytik Jena, Jena, Germany). The immunoblot bands were quantified using VisionWorks LS Image Acquisition and Analysis Software (Analytik Jena), and were expressed as the ratio relative to β-actin protein.

### Statistical analysis

All values are expressed as mean ± SEM. One-way or two-way analysis of variance with repeated measures was used to assess group means, followed by the Scheffé multiple range test for *post hoc* assessment of individual means. *P <* 0.05 was considered statistically significant. It should be mentioned that analysis of the experimental data was carried out in a single-blind manner.

## Results

### Brain infarction after cerebral ischemia

**Fig. 1:**
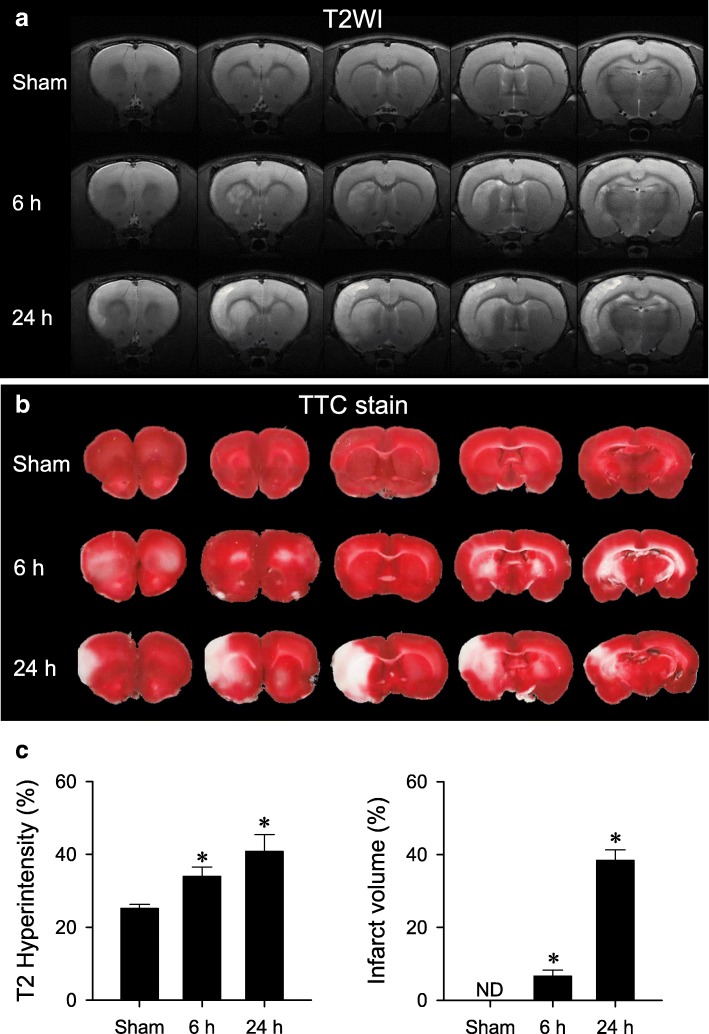
Representative examples of (**a**) T2-weighted coronal imaging (T2WI) and (**b**) TTC-stained coronal sections (2 mm) of the forebrain from sham-controls, or rats 6 h and 24 h after reperfusion from transient middle cerebral artery occlusion (MCAO/R). **c** Temporal changes in hyperintensity measured from T2WI or infarct volume (white tissue) measured from TTC staining in sham-controls or rats 6 h and 24 h after MCAO/R. Values are mean ± SEM, *n* = 4–5 animals per experimental group. *P < 0.05 versus Sham group in the *post hoc* Scheffé multiple-range analysis. ND: below detection limit

T2-weighted coronal images (Fig. 1a) and TTC-stained coronal sections (Fig. 1b) showed that reperfusion after 90 min of MCAO was accompanied by an increase in infarction in the ipsilateral cerebral cortex and striatum. Quantitative analysis of group data (Fig. 1c) showed that significantly augmented infarct volume took place 6 h and 24 h after MCAO/R.

### Increase in Pnn and cleaved caspase-3 expression

**Fig. 2:**
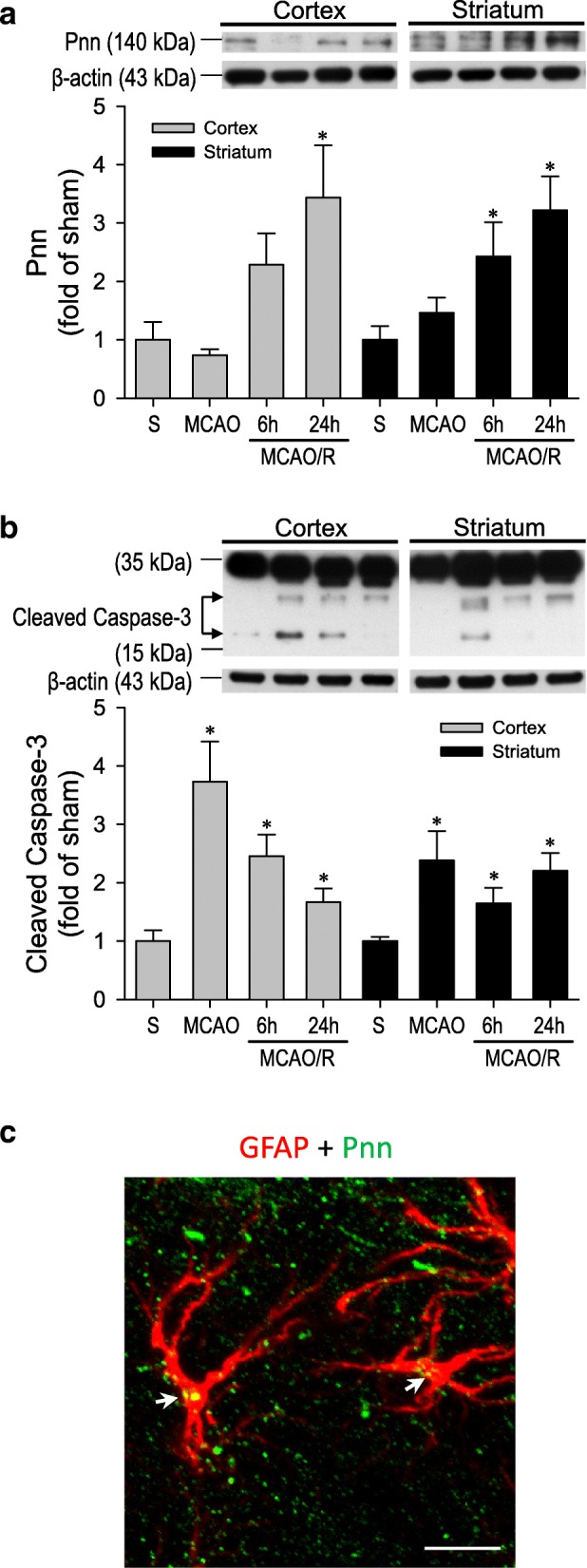
Representative western blot analysis and quantification of temporal fold-changes relative to sham-controls of Pnn (**a**) or cleaved Caspase-3 (**b**) in the ipsilateral cortex and striatum of sham-controls or rats after 90 min of MCAO or 6 h and 24 h after MCAO/R. Values are mean ± SEM of individual samples obtained from 3 to 4 animals per experimental group. *P < 0.05 versus sham-control (S) group in the *post hoc* Scheffé multiple-range analysis. **c** Illustrative example of co-localization (white arrow) of Pnn (green fluorescence) and GFAP (red fluorescence) immunoreactivity in reactive astrocytes at the peri-infarct area of ipsilateral cerebral cortex in rats after MCAO/R. Scale bar: 20 μm

Results from western blot analysis (Fig. 2a) showed that, compared to sham-controls, Pnn protein expression in the peri-infarct area of ipsilateral cortex and striatum exhibited no discernible changes immediately after MCAO, but underwent significant and progressive increases 6 h or 24 h after reperfusion. On the other hand, the protein expression of cleaved caspase-3 (Fig. 2b) in the cytosolic fraction was significantly elevated after 90 min of transient MCAO, followed by a gradual decline over 6 h and 24 h after MCAO/R. Of note is that Pnn immunoreactivity was found to co-localize with a majority of the reactive astrocytes present in the ipsilateral peri-infarct area (Fig. 2c).

### Formation of cytotoxic edema

**Fig. 3:**
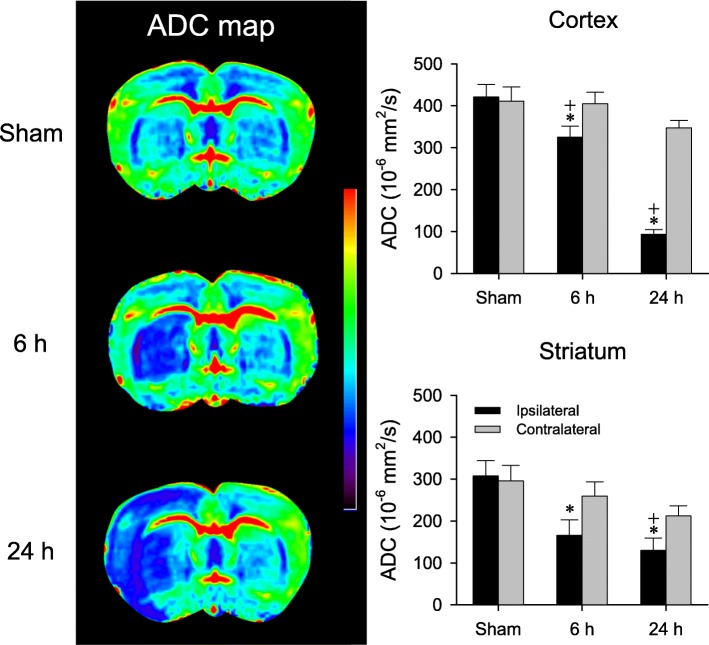
Representative examples of apparent diffusion coefficient (ADC) maps of bilateral cerebral cortex and striatum obtained by diffusion weighted imaging (left panels); or temporal changes in ADC in sham-controls or rats 6 h and 24 h after MCAO/R. Values are mean ± SEM, n = 4–5 animals in each group. *P < 0.05 versus sham-control group, and ^+^P < 0.05 versus measurement from the contralateral side at corresponding time-points in the *post hoc* Scheffé multiple-range analysis

It is generally contended that a decrease of ADC in DWI evaluation denotes formation of cytotoxic edema [34]. Our results revealed a progressive and significant decline in ADC (Fig. 3) in the ipsilateral cortex and striatum 6 h and 24 h after transient MCAO. In contrast, there was much less and insignificant reduction of ADC on the contralateral side at the same MCAO/R time-points.

### Augmented number of reactive astrocytes

**Fig. 4:**
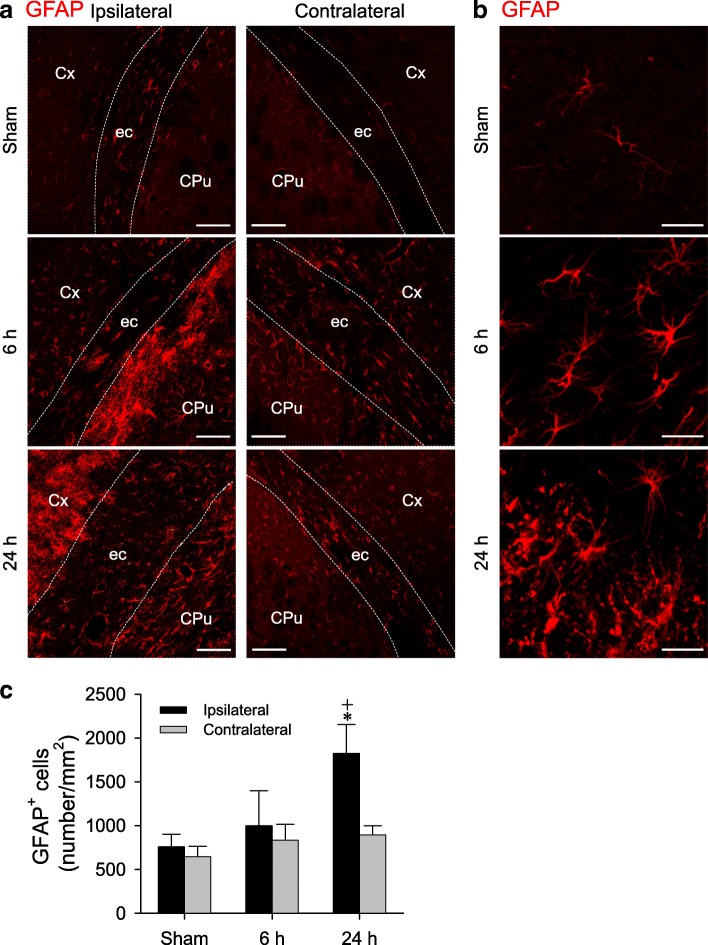
Representative laser scanning confocal microscopic images showing low-power (**a**) and high-power (**b**) views of reactive GFAP-immunoreactive astrocytes at the bilateral (a) or ipsilateral (b) cerebral cortex and striatum in sham-controls or rats 6 h and 24 h after MCAO/R. Scale bars: 100 μm in (**a**) and 20 μm in (**b**). (**c**) Number of GFAP-immunoreactive astrocytes determined by Image J on areas of the bilateral cerebral cortex and striatum represented by (**a**). Values are mean ± SEM, n = 3 animals in each group. *P < 0.05 versus sham-control group, and ^+^*P* < 0.05 versus measurement from the contralateral side at corresponding time-points in the *post hoc* Scheffé multiple-range analysis. Cx, cortex; CPu, corpus striatum putaman; ec, external capsule

It is also generally maintained that astrocytes play a pivotal role in edema formation [35, 36]. Immunofluorescence staining (Fig. 4a) showed that GFAP-immunoreactive cells in the peri-infarct area of ipsilateral cortex and striatum that exhibited the typical features of reactive astrocytes (Fig. 4b) underwent progressively an augmentation in number 6 h and 24 h after cerebral ischemia (Fig. 4c). Comparable changes, albeit to a lesser extent, were observed on the contralateral side.

### Upregulated Pnn augmented Bcl-2 expression in astrocytes after oxygen-glucose deprivation and re-oxygenation

To further interrogate the specific role of Pnn in the engagement of astrocytes in cerebral ischemia implicated by results from the MCAO and MCAO/R experiments, we employed rat primary astrocytes in conjunction with the OGD and OGD/R to mimic the hypoxic-ischemic condition [37]. Western blot analysis indicated that the protein expression of Pnn was significantly increased in primary astrocytes after 24 h of OGD exposure and after 24 h of re-oxygenation (Additional file 5: Figure S4).

**Fig. 5:**
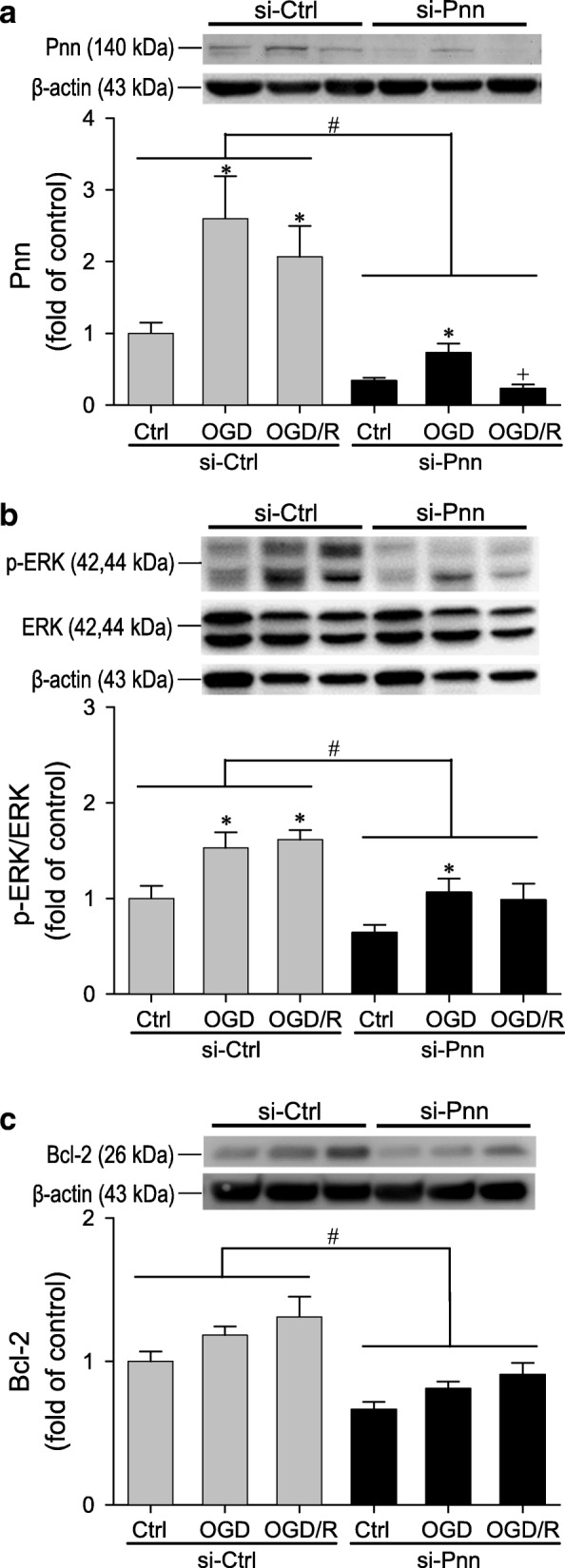
Representative western blot analysis and quantification of temporal fold-changes relative to nonspecific (si-Ctrl) controls of Pnn (**a**), phospho-ERK/ERK (**b**) or Bcl-2 (**c**) expression in rat primary cultured astrocytes under normoxic conditions, after 24 h of exposure to oxygen-glucose deprivation (OGD), or after 24 h of re-oxygenation (OGD/R); and additionally received treatment with *Pnn* specific siRNA (si-Pnn). Values are mean ± SEM of 5–6 independent experiments. *P < 0.05 versus normoxic control (Ctrl) group, ^+^P < 0.05 versus OGD group, and ^#^P < 0.05 versus nonspecific siRNA group (si-Ctrl) in the *post hoc* Scheffé multiple-range analysis

We then employed gene-knockdown to ascertain a causal cellular role for astrocytic Pnn during OGD and OGD/R. In preliminary trials to establish the effective concentration and treatment time, we found that transfection with *Pnn* specific siRNA (si-Pnn) at a final concentration of 10 nM induced significant decrease in Pnn mRNA or protein levels in primary astrocytes 48 h after application (Additional file 3: Figure S2) without significantly affecting cell survival when compared to non-specific siRNA (si-Ctrl) (93.36 ± 3.55% versus 100 ± 2.39%; *n* = 4) based on WST-1 cell viability assay). Under this treatment scheme (Fig. 5a), si-Pnn significantly reduced the Pnn protein level of primary astrocytes in the normoxic control group and blunted the augmented Pnn expression after OGD and OGD/R. Western blot analysis further demonstrated that compared to si-Ctrl group, *Pnn* knockdown significantly blunted the activation of ERK signaling (Fig. 5b) at a time-course that correlated to the reduction of Bcl-2 protein levels in astrocytes in the normoxic control group and after OGD and OGD/R (Fig. 5c). We also observed an increase of cleaved caspase-3 in primary cultures of astrocyte under normoxia and OGD treatment, of which was significantly augmented by treatment with Pnn siRNA when compared to treatment with non-specific siRNA (Additional file 6: Figure S5).

### Pnn sustains mitochondrial bioenergetics in astrocytes

**Fig. 6:**
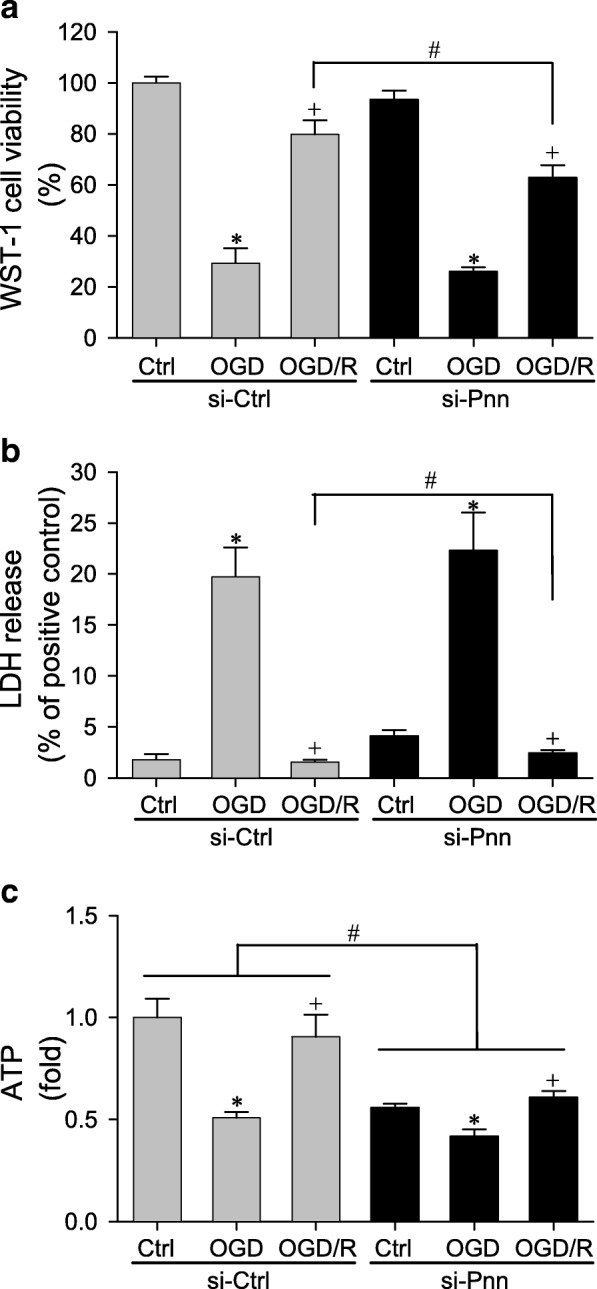
Effects of treatment with *Pnn* specific siRNA (si-Pnn) or control nonspecific siRNA (si-Ctrl) on cell proliferation measured by the WST-1 assay (**a**), LDH leakage (**b**), and fold changes relative to si-Ctrl group of cellular ATP level (**c**) in rat primary astrocytes under normoxic conditions, after 24 h of exposure to oxygen-glucose deprivation (OGD), or after 24 h of re-oxygenation (OGD/R). Values are mean ± SEM of 3–4 independent experiments. *P < 0.05 versus normoxic control (Ctrl) group, ^+^P < 0.05 versus OGD group, and ^#^P < 0.05 versus nonspecific siRNA group (si-Ctrl) in the *post hoc* Scheffé multiple-range analysis

In primary astrocytes treated with si-Ctrl, OGD significantly reduced cell viability (Fig. 6a), elevated LDH level in medium (Fig. 6b), and decreased ATP production (Fig. 6c). All these changes were significantly reversed after OGD/R. Knockdown of *Pnn* retarded the recovery of primary astrocytes from the reduction of cell viability (Fig. 6a). Treatment with si-Pnn additionally increased LDH release (Fig. 6b) and reduced ATP levels (Fig. 6b) in the normaxic control group; exacerbated the decrease in ATP level in the OGD group, and impeded the reversal from decline in cell viability (Fig. 6a), elevation in LDH leakage (Fig. 6b) and decrease in ATP production (Fig. 6c) in the OGD/R group.

### Pnn protects astrocytes from OGD-induced cell death

**Fig. 7:**
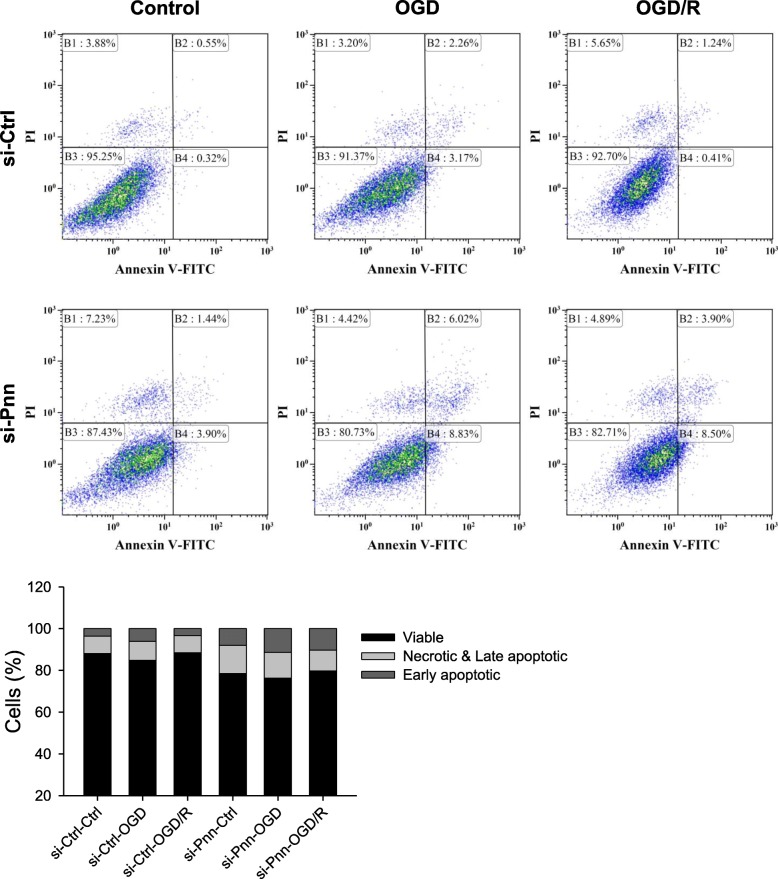
Representative dot plot of annexin V- propidium iodide (PI) staining from flow cytometric analysis (upper panels) and quantification by stacked bar graph (lower panel) showing the effects of treatment with *Pnn* specific siRNA (si-Pnn) or control nonspecific siRNA (si-Ctrl) on types of cell death in rat primary astrocytes under normoxic conditions, after 24 h of exposure to oxygen-glucose deprivation (OGD), or after 24 h of re-oxygenation (OGD/R)

Flow cytometry (Fig. 7) showed that treatment with *Pnn* siRNA enhanced the proportions of early apoptotic cells (annexin V^+^/PI^−^) and necrotic & late apoptotic cells (annexin V^−^/PI^+^ & annexin V^+^/PI^+^) in the control and OGD or OGD/R groups when compared to the si-Ctrl group. Under si-Pnn treatment, the proportions of live (annexin V^−^/PI^−^) cells were accordingly reduced in all three groups.

## Discussion

Previous studies on Pnn focused on epithelial cell-cell adhesion, cancer progression, and pre-mRNA splicing in embryonic development [6–11]. Based on corroborative observations from MCAO animal model and OGD cell model of ischemic stroke, the present study provided the first documentation that Pnn also protects astrocytes from apoptotic and necrotic cell death induced by ischemia via maintenance of mitochondrial anti-apoptotic (Bcl-2 upregulation) and bioenergetics functions.

Our first piece of evidence that implicates a role for Pnn in ischemic stroke arises from the significant increase in its expression at the peri-infarct area in the ipsilateral cortex and striatum 6 h and 24 h after transient MCAO, which correlates positively with the time-course of the induced infarction detected by T2WI and TTC stain. By showing that the temporal pattern of Pnn upregulation also paralleled that exhibited by the increase in the number of reactive astrocytes at the same cortical and striatal areas, our second piece of evidence in effect links Pnn specifically to the engagement of astrocytes in ischemic stroke. This link is further reinforced by the finding that Pnn immunoreactivity co-localized with GFAP-positive reactive astrocytes in the peri-infarct area. Although it is beyond the scope of this study, we wish to stress that since western blot analysis using brain tissue samples may not reveal the exclusive changes of Pnn protein levels in astrocytes, and our immunofluorescence results also showed positive Pnn-immunoreactivity that was not co-localized with GFAP, the possibility exists for the involvement of other cell types in the cortical and striatal areas in the engagement of Pnn in ischemia stroke.

Unlike vasogenic edema that accompanies the breakdown of blood-brain barrier, cytotoxic edema represents simply the redistribution of water from extracellular to intracellular compartments. As cells swell because of an inward shift of water, there is a commensurate decrease in diffusion, hence the lowered signal for ADC [34]. It is generally contended that cytotoxic edema is the result of cells being unable to maintain ATP-dependent sodium/potassium pumps in the cell membrane due either to hypoxia or ischemia [38]. As such, our DWI results showing that the progressive reduction in ADC during MCAO/R mirrored the elevation of hyperintensity measured by T2WI are of interests. The implied occurrence of cytotoxic edema that accompanies reperfusion after MCAO hints that bioenergetics failure underpins ischemic stroke. These DWI and T2WI data, however, did not offer direct information on the engagement of astrocytes in this process. However, given the relationship between astrocytes and edema formation after ischemic stroke [35, 36], the participation of reactive astrocytes in this process is anticipated and indeed confirmed in subsequent in vitro studies, along with a causal role for Pnn.

With the interposing neurons, astrocytes, microglia, oligodendrocytes and blood vessels as the constitution of the brain, it is impossible to decipher the specific contribution of the astrocytes in ischemic stroke using the MCAO and MCAO/R model. We therefore employed rat primary cultured astrocytes in conjunction with the OGD and OGD/R model in a series of proof-of-principle experiments to document the specific neuroprotective role of astrocytes under ischemic conditions and delineate the causal and mechanistic contributions of Pnn in this process. We reasoned that measurements from the approximately 20% of primary astrocytes that survived OGD and those that amounted to almost 80% of the normoxic control group after OGD/R may be taken as the equivalent of results obtained from astrocytes that remain after MCAO and the proliferated reactive astrocytes that are present during MCAO/R in the peri-infarct areas. Based on this interpretation, we first established the link between Pnn and astrocytes in the peri-infarct areas of the cortex and striatum identified from our animal model of ischemic stroke by demonstrating that primary astrocytes also exhibited upregulation of Pnn in the OGD and OGD/R groups. Subsequent experiments employing gene-knockdown revealed that Pnn reduces astrocytic necrotic and apoptotic cell death via maintenance of mitochondrial anti-apoptotic and bioenergetics functions.

An anti-apoptotic role for Pnn in ischemic stroke is implicated by the observation that the increase in Pnn expression during MCAO and MCAO/R mirrored the decrease in cleaved caspase-3 at the peri-infarct area in cortex and striatum. Western blot analysis further demonstrated that *Pnn* knockdown significantly augmented the already increased cleaved caspase-3 in astrocytes after OGD treatment. Thus, it is intriguing that results from our flow cytometric analyses showed that treatment with si-Pnn more than doubled the chance of the otherwise living primary astrocytes to manifest apoptotic and necrotic cell death under conditions of normoxia, OGD or OGD/R. This suggested intrinsic anti-apoptotic action of the endogenous Pnn is supported by the observation that si-Pnn treatment similarly reduced Bcl-2 expression under all three experimental conditions. As a key component of the mitochondrial anti-apoptotic machinery [39], Bcl-2 acts on the mitochondrial membrane to inhibit permeability and release of the mitochondrial apoptogenic factors, including cytochrome c and apoptosis-inducing factor; and prevent the activation of caspases through sequestering pro-caspases. Upregulation of Bcl-2 in brain tissue with ischemic injury is thought to play an important role in post-stroke neuroprotection [40, 41]. It follows that Pnn may elicit its neuroprotective action on the reactive astrocytes in the peri-infarct area after ischemic stroke via maintenance of mitochondrial anti-apoptotic functions. Previous studies showed that the expression level of Pnn is associated with ERK signaling-mediated stress response to glucose deprivation in cancer cells [10, 11]; and activation of ERK signaling mediates Bcl-2 upregulation in various cell types [42, 43]. Together with our observations that *Pnn* knockdown significantly blunted the activation of ERK signaling that was temporally correlated to the reduction of Bcl-2 protein levels in astrocytes in the normoxic control group and after OGD and OGD/R, it is conceivable that ERK signaling may act as the intermediate between the anti-apoptotic effects of Pnn and upregulation of Bcl-2 in astrocytes against ischemic stress.

The occurrence of cytotoxic edema that accompanies reperfusion after MCAO also implicates that bioenergetics failure may underpin ischemic stroke. The significant decrease in cell viability demonstrated by the WST-1 assay indicative of declined mitochondrial dehydrogenase activity and the increase in LDH released into the medium indicative of mitochondrial malfunction; alongside the reduction in ATP levels amply ascertained that mitochondrial bioenergetics failure indeed took place in primary astrocytes during OGD. A protective role for the endogenous Pnn is again demonstrated when *Pnn* gene-knockdown enhanced LDH leakage and ATP reduction in the normoxic control group. This is reinforced by the observation that si-Pnn treatment impeded the reversal from decline in cell viability, elevation in LDH release and decrease in ATP production in the OGD/R group. It follows that Pnn may elicit its neuroprotective action on the astrocytes in the peri-infarct area after ischemic stroke via maintenance of mitochondrial bioenergetics functions.

In response to ischemia, astrocytes produce multiple neurotrophic factors to protect neuron [21], including brain-derived neurotrophic factor [44], glia-derived neurotrophic factor [44, 45]; nerve growth factor [46], and vascular endothelia growth factor [47]. Wei et al. [10] demonstrated that Pnn facilitates cell proliferation through activating epidermal growth factor receptor signaling pathway. Whether the protective actions of Pnn entail those astrocyte-derived neurotrophic factors remain to be established.

## Conclusion

Cerebral stroke remains one of the leading causes of death world-wide, and ischemic stroke caused by sudden loss of blood flow in brain is the major form of cerebral stroke. One evolving concept over the last three decades is the employment of the ischemic peri-infarct area as the target for neuroprotection [48], with astrocytes assuming a pivotal role [21–24]. Based on corroborative results obtained from MCAO and MCAO/R in rats and OGD and OGD/R in primary cultured astrocytes, the present study provided the first demonstration that the endogenous Pnn participates in this protective process by preserving the viability of the astrocytes that survived the ischemic challenge via maintenance of mitochondrial anti-apoptotic and bioenergetics functions.

## Additional files

Additional file 1: Supplemental materials and methods. (DOCX 24 kb)

Additional file 2: **Figure S1**. Diagrammatic representation of methods to calculate hyperintensity in T2WI (a) and TTC-stained infarct volume (b) after MCAO. (DOCX 373 kb)

Additional file 3: **Figure S2**. (a) Fold-changes relative to control group of *Pnn* mRNA expression in rat primary astrocytes detected at different time-points (24 or 48 h) after treatment with different concentrations (10 or 20 nM) of *Pnn* specific siRNA (si-Pnn). Values are mean ± SEM of 3 independent experiments. *P < 0.05 versus control (Ctrl) group in the post hoc Scheffé multiple-range analysis. (b) Representative western blots (insets) of Pnn relative to β-actin in rat primary astrocytes detected at different time-points (24 or 48 h) after treatment with different concentrations (10 or 20 nM) of *Pnn* specific siRNA (si-Pnn). (DOCX 145 kb)

Additional file 4: **Figure S3.** Determination of cell death status of astrocytes using flow cytometry. Rat primary astrocytes were harvested and stained with annexin V-FITC and propidium iodide (PI). Astrocytes were gated based on light-scattering properties in the SS and FS modes (A). 10,000 events per sample within gate A were collected. Three controls including unstained cells, cells stained with annexin V-FITC (alone), and cells stained with PI (alone) were executed to set up compensation and quadrants. Data were analyzed by Kaluza software (Beckman Coulter). (DOCX 218 kb)

Additional file 5: **Figure S4.** Representative western blot and quantitative analysis of fold-changes relative to normoxic control group of Pnn expression in rat primary astrocytes subjected to oxygen-glucose deprivation (OGD) or re-oxygenation (OGD/R). Values are mean ± SEM of 4–5 independent experiments. *P < 0.05 versus normoxic control (Ctrl) group in the *post hoc* Scheffé multiple-range analysis. (DOCX 35 kb)

Additional file 6: **Figure S5.** Representative western blot analysis and quantification of temporal fold-changes relative to nonspecific (si-Ctrl) controls of cleaved caspase-3 expression in rat primary cultured astrocytes under normoxic conditions, after 24 h of exposure to oxygen-glucose deprivation (OGD), or after 24 h of re-oxygenation (OGD/R); and additionally received treatment with *Pnn* specific siRNA (si-Pnn). Values are mean ± SEM of 4–5 independent experiments. *P < 0.05 versus normoxic control (Ctrl) group, ^+^P < 0.05 versus OGD group, and ^#^P < 0.05 versus nonspecific siRNA group (si-Ctrl) in the *post hoc* Scheffé multiple-range analysis. (DOCX 226 kb)

## Data Availability

The datasets used and/or analyzed during the current study are available from the corresponding author on reasonable request.

## Abbreviations

ADC: Apparent diffusion coefficient
CCA: Common carotid artery
DMEM: Dulbecco’s modified Eagle medium
DWI: Diffusion weighted imaging
FBS: Fetal bovine serum
GFAP: Glial fibrillary acidic protein
HRP: Horseradish peroxidase
LDH: Lactate dehydrogenase
MCAO and MCAO/R: Middle cerebral artery occlusion and reperfusion
MEM: Minimum essential media
MRI: Magnetic resonance imaging
OGD and OGD/R: Oxygen-glucose deprivation and re-oxygenation
P1: Postnatal day 1
PBS: Phosphate-buffered saline
PI: Propidium iodide
Pnn: Pinin
PVDF: Polyvinylidenedifluoride
T2WI: T2-weighted imaging
TTC: 2,3,5-Triphenyltetrazolium chloride
