# Supplementary material for: Pinin protects astrocytes from cell death after acute ischemic stroke via maintenance of mitochondrial anti-apoptotic and bioenergetics functions
Source: J Biomed Sci. 2019 Jun 5;26:43. doi: 10.1186/s12929-019-0538-5 (PMC6549339; doi:10.1186/s12929-019-0538-5)
Supplement: Supplementary file 1 — Supplemental materials and methods. (DOCX 24 kb) [file 12929_2019_538_MOESM1_ESM.docx]

**Supplementary Materials and Methods**

***Animal model of focal cerebral ischemia***

We used the middle cerebral artery occlusion (MCAO) method to induce acute focal cerebral ischemia, using published surgical procedures [1-4] with modification. Adult male Sprague-Dawley rats (12 weeks old) were anesthetized with 2% isoflurane and immobilized in the supine position on a surgical table. Under the operating microscope, a longitudinal cervical midline incision (approximately 2 cm) was made and the superficial fascia was dissected. The left common carotid artery (CCA) was carefully isolated from the vagus nerve and ligated with a sterile 4-0 silk suture by a single knot, 6-7 mm caudal to the bifurcation of the CCA into the external carotid artery (ECA) and internal carotid artery (ICA). The ECA and the occipital artery (OA) originating as the first branch of ECA was typically ligated permanently. Another silk suture was tied loosely around the ICA. A microvascular clamp was placed on the ICA front of the silk suture. A punctate incision was made in the ventral wall of the CCA with a 25G needle. A nylon monofilament suture (RWD Life Science; Shenzhen, China), 5 cm in length with a silicone-rubber coated tip (0.43 ± 0.02 mm diameter), was advanced into the CCA lumen towards the microvascular clamp. The silk suture around the ICA was tightened to prevent bleeding, and the microvascular clamp was removed from the ICA. This was followed by advancing the nylon monofilament suture from the ICA towards the middle cerebral artery (MCA), typically 20 mm in length. At this point, the timer was started to record the occlusion time. After 90 min, the ICA suture was loosened and the nylon monofilament suture was withdrawn to the CCA. The microvascular clamp was placed on the ICA, the suture was removed; and the incision in CCA was covered using cyanoacrylate glue (3M Vetbond Adhesive; World Precision Instruments, Hitchin, UK). The microvascular clamp and CCA knot were carefully removed and perfusion through the CCA was verified. The end of occlusion (reperfusion start) time was recorded, and the wound closed. Sodium penicillin (10,000 IU; YF Chemical, Taiwan) was given intramuscularly to prevent postoperative infection. As a routine, the effects of 90-min transient MCAO, and 6 h or 24 h after reperfusion (MCAO/R) were evaluated in this study. Animals that received the same surgical procedures under isoflurane anesthesia but without MCAO served as the sham controls.

***Magnetic resonance imaging (MRI)***

We carried out sequential MRI acquisition in rats anesthetized with isoflurane using a 9.4T horizontal-bore animal MR scanning system (Biospec 94/20; Bruker, Ettingen, Germany) before and at 6 h and 24 h after 90 min transient MCAO to examine brain infarction and edema.

T2-weighted coronal imaging (T2WI) was acquired using multislice turbo rapid acquisition with refocusing echoes (Turbo-RARE) sequence using the following parameters: field of view (FOV) = 20.0 × 20.0 mm; matrix dimension = 256 × 256 pixels; spatial resolution/pixel = 78 × 78 μm; slice thickness = 1 mm; interslice distance= 1 mm; effective echo time (TE)= 28 ms; echo time = 9.33 ms; repetition time (TR) = 3000 ms; rare factor = 8; refocusing flip angle = 180 deg; number of averages = 9; number of repetitions (NR) = 1; total acquisition time = 14 min 24 s. The infarct zone appears hyperintense (bright) on T2 images. We used ImageJ version 1.48v to quantify the hyperintense area of T2 image.

Diffusion weighted imaging (DWI) was acquired using DtiEpi sequence on the same spatial dimension as in the T2-weighted coronal imaging. The parameters used for acquisition were: FOV = 20 mm × 20 mm; matrix dimension = 96 × 96 pixels; spatial resolution/pixel= 208 μm × 208 μm; slice thickness= 1 mm; echo time = 20 ms; TR = 3800 ms; optimized b value/direction= 100, 300, 500, 700, 800, 1000 s/mm^2^; number of b0 images= 1; gradient duration= 2.5 ms; gradient separation= 8 ms; number of segments = 2; number of averages = 36; acquisition time= 31 min 55 s. ParaVision 5.1 software (Bruker) and MIstar (ver. 3.2.63; Apollo Medical Imaging Technology, Melbourne, Australia) were applied to process the DWI and apparent diffusion coefficient (ADC) maps respectively.

***Quantification of volume of T2WI hyperintensity***

Quantification of the hyperintense area of T2WI were performed with a limited threshold (**Figure S1a**). Images were converted into 8-bit grey scale images and the integrated density (IntDen) was measured using ImageJ version 1.48v. Hyperintensity (A %) = IntDen of hyperintense area (A)/IntDen of half-brain (A + B) × 100 %.

***Isolation of RNA and real-time PCR***

Total RNA from primary astrocytes was isolated with a Quick-RNA kit (Zymo Research, Irvine, CA, USA) according to the manufacturer’s instructions. All RNA isolated was quantified by spectrophotometry and the optical density 260/280 nm ratio was determined. Reverse transcriptase reaction was performed using a PrimeScript RT Reagent Kit (Takara, Shiga, Japan). *Pnn* and *GAPDH* gene expression was quantified using TaqMan probe (product no. Rn01498866_g1 and Rn01775763_g1 respectively) and StepOnePlus Real-time PCR system (Applied Biosystems, Foster City, CA, USA). Relative gene expression was calculated by the 2^-∆∆Ct^ method.

***Flow cytometry***

To determine cell death status after OGD and/or re-oxygenation, primary astrocytes were harvested and washed, and then stained with the eBioscience™ Annexin V-FITC Apoptosis Detection Kit (Invitrogen, Carlsbad, CA, USA). Annexin V- and propidium iodide (PI)-double negative cells were classified as viable, annexin V-positive and PI-negative as early apoptotic, annexin V- and PI-positive cells as late apoptotic, and annexin V-negative and PI-positive cells as necrotic. Stained cells were analyzed by Gallios™ Flow Cytometer (Beckman Coulter, Indianapolis, IN, USA). In brief, primary astrocytes were harvested using 0.1 % trypsin and resuspended with medium and centrifuged at 420 x g for 5 min. Cells were washed twice with 1X PBS twice and once with 1X Binding Buffer. The fluorochrome-conjugated Annexin V (5 μl) was added to 100 μl of the cell suspension and incubated for 15 min at room temperature. Cells were then washed in 1X Binding Buffer and resuspended in 200 μl of 1X Binding Buffer. The propidium iodide (PI) staining buffer (5 μl) was added and stood for 5 min in the dark. After adding 300 μl of 1X Binding Buffer to each tube, stained cells were transferred to round bottom tubes and were analyzed by flow cytometry. Data were analyzed by Kaluza software (Beckman Coulter).

**References**

1. Uluç K, Miranpuri A, Kujoth GC, Aktüre E, Başkaya MK. Focal cerebral ischemia model by endovascular suture occlusion of the middle cerebral artery in the rat. J Vis Exp. 2011;48:1978.
2. Güzel A, Rölz R, Nikkhah G, Kahlert UD, Maciaczyk J. A microsurgical procedure for middle cerebral artery occlusion by intraluminal monofilament insertion technique in the rat: a special emphasis on the methodology. Exp Transl Stroke Med. 2014;6:6.
3. Hill JW, Nemoto EM. Transient middle cerebral artery occlusion with complete reperfusion in spontaneously hypertensive rats. MethodsX. 2014;1:283-291.
4. Lee S, Lee M, Hong Y, Won J, Lee Y, Kang SG, et al. Middle cerebral artery occlusion methods in rat versus mouse models of transient focal cerebral ischemic stroke. Neural Regen Res. 2014;9:757-758.
