# Supplementary material for: Pinin protects astrocytes from cell death after acute ischemic stroke via maintenance of mitochondrial anti-apoptotic and bioenergetics functions
Source: J Biomed Sci. 2019 Jun 5;26:43. doi: 10.1186/s12929-019-0538-5 (PMC6549339; doi:10.1186/s12929-019-0538-5)
Supplement: Supplementary file 2 — Figure S1. Diagrammatic representation of methods to calculate hyperintensity in T2WI (a) and TTC-stained infarct volume (b) after MCAO. (DOCX 373 kb) [file 12929_2019_538_MOESM2_ESM.docx]

**
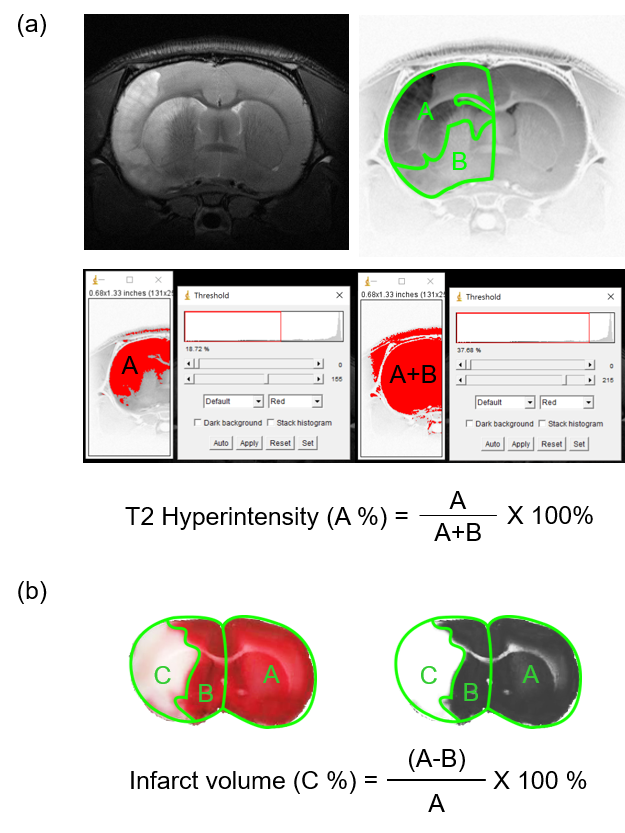
**

**Figure S1.** Diagrammatic representation of methods to calculate hyperintensity in T2WI (a) and TTC-stained infarct volume (b) after MCAO.
