# Supplementary material for: Pinin protects astrocytes from cell death after acute ischemic stroke via maintenance of mitochondrial anti-apoptotic and bioenergetics functions
Source: J Biomed Sci. 2019 Jun 5;26:43. doi: 10.1186/s12929-019-0538-5 (PMC6549339; doi:10.1186/s12929-019-0538-5)
Supplement: Supplementary file 3 — Figure S2. (a) Fold-changes relative to control group of Pnn mRNA expression in rat primary astrocytes detected at different time-points (24 or 48 h) after treatment with different concentrations (10 or 20 nM) of Pnn specific siRNA (si-Pnn). Values are mean ± SEM of 3 independent experiments. *P < 0.05 versus control (Ctrl) group in the post hoc Scheffé multiple-range analysis. (b) Representative western blots (insets) of Pnn relative to β-actin in rat primary astrocytes detected at different time-points (24 or 48 h) after treatment with different concentrations (10 or 20 nM) of Pnn specific siRNA (si-Pnn). (DOCX 145 kb) [file 12929_2019_538_MOESM3_ESM.docx]

**
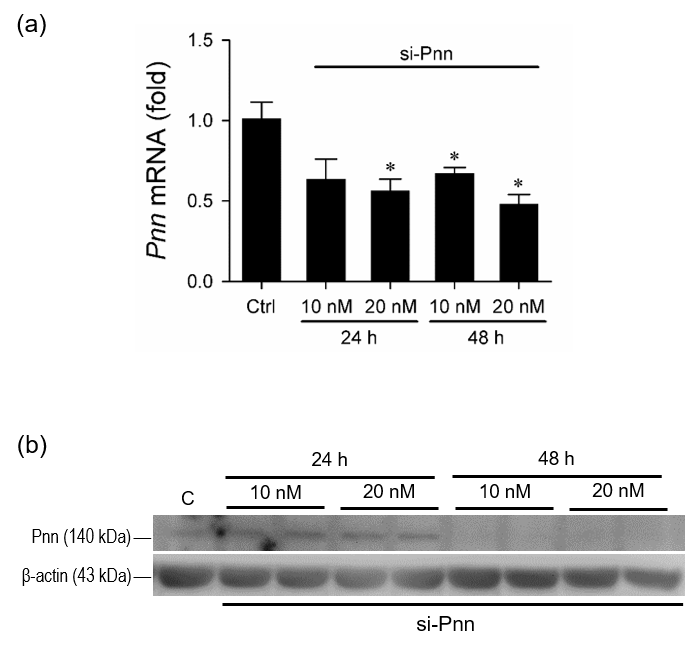
**

**Figure S2.** (a) Fold-changes relative to control group of *Pnn* mRNA expression in rat primary astrocytes detected at different time-points (24 or 48 h) after treatment with different concentrations (10 or 20 nM) of Pnn specific siRNA (si-Pnn). Values are mean ± SEM of 3 independent experiments. *P < 0.05 versus control (Ctrl) group in the post hoc Scheffé multiple-range analysis. (b) Representative western blots (insets) of Pnn relative to β-actin in rat primary astrocytes detected at different time-points (24 or 48 h) after treatment with different concentrations (10 or 20 nM) of Pnn specific siRNA (si-Pnn).
