# Supplementary material for: Pinin protects astrocytes from cell death after acute ischemic stroke via maintenance of mitochondrial anti-apoptotic and bioenergetics functions
Source: J Biomed Sci. 2019 Jun 5;26:43. doi: 10.1186/s12929-019-0538-5 (PMC6549339; doi:10.1186/s12929-019-0538-5)
Supplement: Supplementary file 4 — Figure S3. Determination of cell death status of astrocytes using flow cytometry. Rat primary astrocytes were harvested and stained with annexin V-FITC and propidium iodide (PI). Astrocytes were gated based on light-scattering properties in the SS and FS modes (A). 10,000 events per sample within gate A were collected. Three controls including unstained cells, cells stained with annexin V-FITC (alone), and cells stained with PI (alone) were executed to set up compensation and quadrants. Data were analyzed by Kaluza software (Beckman Coulter). (DOCX 218 kb) [file 12929_2019_538_MOESM4_ESM.docx]

**
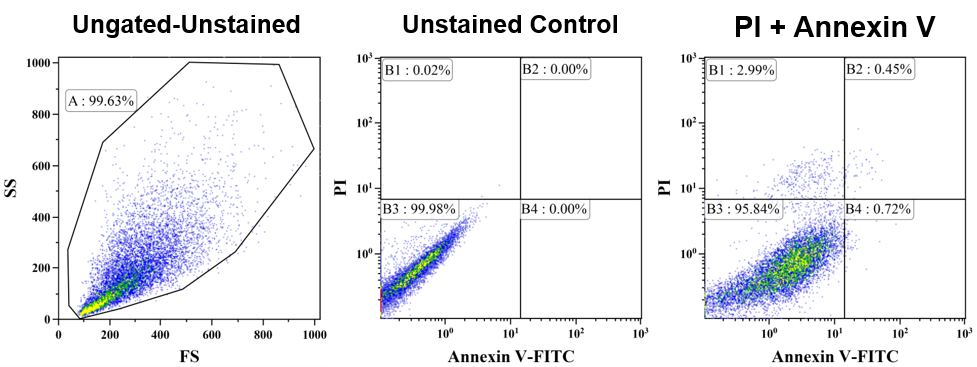
**

**Figure S3.** Estimation of cell death status of astrocytes using flow cytometry. Rat primary astrocytes were harvested and stained with the annexin V-FITC and propidium iodide (PI). Astrocytes were gated based on light-scattering properties in the SS and FS modes (A). 10,000 events per sample within the gate A were collected. Three controls including unstained cells, cells stained with annexin V-FITC (alone), and cells stained with PI (alone) were executed to set up compensation and quadrants. Data were analyzed by Kaluza software (Beckman Coulter).
