# Supplementary material for: Pinin protects astrocytes from cell death after acute ischemic stroke via maintenance of mitochondrial anti-apoptotic and bioenergetics functions
Source: J Biomed Sci. 2019 Jun 5;26:43. doi: 10.1186/s12929-019-0538-5 (PMC6549339; doi:10.1186/s12929-019-0538-5)
Supplement: Supplementary file 5 — Figure S4. Representative western blot and quantitative analysis of fold-changes relative to normoxic control group of Pnn expression in rat primary astrocytes subjected to oxygen-glucose deprivation (OGD) or re-oxygenation (OGD/R). Values are mean ± SEM of 4–5 independent experiments. *P < 0.05 versus normoxic control (Ctrl) group in the post hoc Scheffé multiple-range analysis. (DOCX 35 kb) [file 12929_2019_538_MOESM5_ESM.docx]

**
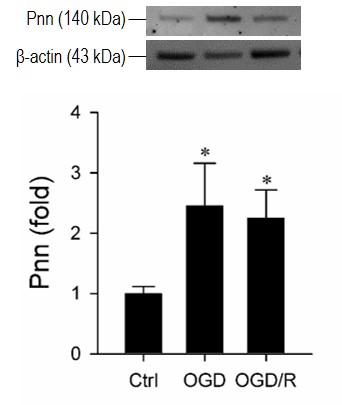
**

**Figure S4.** Representative western blot and quantitative analysis of fold-changes relative to normoxic control group of Pnn expression in rat primary astrocytes subjected to oxygen-glucose deprivation (OGD) or re-oxygenation (OGD/R). Values are mean ± SEM of 4-5 independent experiments. *P < 0.05 versus normoxic control (Ctrl) group in the post hoc Scheffé multiple-range analysis.
