# Supplementary material for: Pinin protects astrocytes from cell death after acute ischemic stroke via maintenance of mitochondrial anti-apoptotic and bioenergetics functions
Source: J Biomed Sci. 2019 Jun 5;26:43. doi: 10.1186/s12929-019-0538-5 (PMC6549339; doi:10.1186/s12929-019-0538-5)
Supplement: Supplementary file 6 — Figure S5. Representative western blot analysis and quantification of temporal fold-changes relative to nonspecific (si-Ctrl) controls of cleaved caspase-3 expression in rat primary cultured astrocytes under normoxic conditions, after 24 h of exposure to oxygen-glucose deprivation (OGD), or after 24 h of re-oxygenation (OGD/R); and additionally received treatment with Pnn specific siRNA (si-Pnn). Values are mean ± SEM of 4–5 independent experiments. *P < 0.05 versus normoxic control (Ctrl) group, +P < 0.05 versus OGD group, and #P < 0.05 versus nonspecific siRNA group (si-Ctrl) in the post hoc Scheffé multiple-range analysis. (DOCX 226 kb) [file 12929_2019_538_MOESM6_ESM.docx]

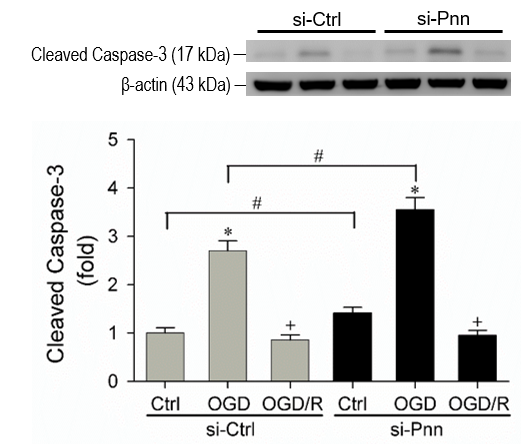


**Figure S5.** Representative western blot analysis and quantification of temporal fold-changes relative to nonspecific (si-Ctrl) controls of cleaved caspase-3 expression in rat primary cultured astrocytes under normoxic conditions, after 24 h of exposure to oxygen-glucose deprivation (OGD), or after 24 h of re-oxygenation (OGD/R); and additionally received treatment with *Pnn* specific siRNA (si-Pnn). Values are mean ± SEM of 4-5 independent experiments. *P < 0.05 versus normoxic control (Ctrl) group, ^+^P < 0.05 versus OGD group, and ^#^P < 0.05 versus nonspecific siRNA group (si-Ctrl) in the post hoc Scheffé multiple-range analysis.
